# mIoT: Metamorphic IoT Platform for On-Demand Hardware Replacement in Large-Scaled IoT Applications

**DOI:** 10.3390/s20123337

**Published:** 2020-06-12

**Authors:** Dongkyu Lee, Hyeongyun Moon, Sejong Oh, Daejin Park

**Affiliations:** 1 School of Electronics Engineering, Kyungpook National University, Daegu 41566, Korea; dklee1215@knu.ac.kr (D.L.); moonhg1209@gmail.com (H.M.); 2 Nvidia Corporation, Santa Clara, CA 95051, USA; Sejongo@nvidia.com

**Keywords:** fault safe, reconfigurable hardware, RISC-V, Internet of Things (IoT), edge computing

## Abstract

As the Internet of Things (IoT) is becoming more pervasive in our daily lives, the number of devices that connect to IoT edges and data generated at the edges are rapidly increasing. On account of the bottlenecks in servers, due to the increase in data, as well as security and privacy issues, the IoT paradigm has shifted from cloud computing to edge computing. Pursuant to this trend, embedded devices require complex computation capabilities. However, due to various constraints, edge devices cannot equip enough hardware to process data, so the flexibility of operation is reduced, because of the limitations of fixed hardware functions, relative to cloud computing. Recently, as application fields and collected data types diversify, and, in particular, applications requiring complex computation such as artificial intelligence (AI) and signal processing are applied to edges, flexible processing and computation capabilities based on hardware acceleration are required. In this paper, to meet these needs, we propose a new IoT platform, called a metamorphic IoT (mIoT) platform, which can various hardware acceleration with limited hardware platform resources, through on-demand transmission and reconfiguration of required hardware at edges instead of via transference of sensing data to a server. The proposed platform reconfigures the edge’s hardware with minimal overhead, based on a probabilistic value, known as callability. The mIoT consists of reconfigurable edge devices based on RISC-V architecture and a server that manages the reconfiguration of edge devices based on callability. Through various experimental results, we confirmed that the callability-based mIoT platform can provide the hardware required by the edge device in real time. In addition, by performing various functions with small hardware, power consumption, which is a major constraint of IoT, can be reduced.

## 1. Introduction

Internet of Things (IoT) devices are connected electronic devices, vehicles, buildings, and various social infrastructures that communicate with each other and process data in real time. As IoT becomes more popular, the number of connected devices and sensors (we call these “edges”) are sharply increasing, and data are also overflowing from these devices [1,2]. The types of devices are growing more varied and computations to process the data are becoming complicated, according to diverse applications in industry fields [3,4,5]. Thus, the complexity of IoT systems has increased.

Traditionally, these various edge devices have performed such operations as sensing data, controlling target systems and environments, and communicating with servers of IoT systems. Recently, edge devices have come to require the ability to process sensed data [1,6]. However, the edge environment cannot provide sufficient computing power, due to constraints of the edge environment, such as insufficient power supply, unstable operating environments, and insufficient memory size: the edge environment is also demanding in terms of maintenance, involving frequent device changes, firmware updates, and physical access difficulties [7,8,9].

**Figure 1:**
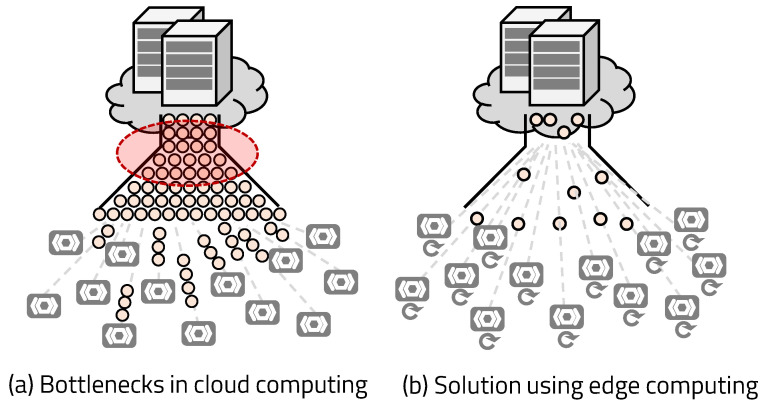
Cloud platform’s bottleneck issues.

Cloud computing is proposed to resolve the slow performance of the edge, which derives from various constraints [10]. Cloud computing can process various data successfully and flexibly using the powerful hardware resources of a server. However, servers used in cloud computing have two constraints: network bandwidth and server workload. As shown in Figure 1, the more devices are connected and the more data is transmitted to the cloud server, the slower the service processing speed due to these constraints, so real-time processing cannot be guaranteed. Recently, with the development of wireless communication, represented by 5G and Bluetooth, and wired communication, represented by optical fiber, the speed of the network has been increased, and the network bottleneck has been significantly reduced. On the other hand, the workload problem due to the limitation of the resources for a server is getting serious. In addition, the entire IoT system is dependent on the network, making it vulnerable to network errors that can cause the entire system to go offlnie [9,11,12].

To reduce network dependency, edge computing, which processes data at a local computer, was introduced. Edge computing processes data on the computer around the edge device, resulting in low network dependency and the ability to use additional hardware accelerators to expedite data processing. However, IoT systems operating in poor environments and small-scale IoT systems cannot use local computers, so certain edge platforms require systems that can process data on their own, such as MCU (Micro Controller Unit)-based platforms. These MCU-based devices use hardware accelerators, which quickly perform complex tasks but have limited work to do, to compensate for the slow processing speed. IoT edge devices require a stable edge system with a flexible hardware accelerator that can perform various services required in various environments in a small hardware space.

Recently released FPGAs (Field Programmable Gate Arrays) are better suited for edge computing, which requires faster processing speeds and flexible accelerators, with small, low-power, low-cost features. The ASIC-FPGA (Application-Specific Integrated Circuit-Field Programmable Gate Arrays) co-design architecture was proposed to take advantage of the small size and low-power characteristics of the ASIC (Application-Specific Integrated Circuit) and the flexibility of the FPGA [13,14,15]. The functional hardware blocks are partitioned into the ASIC and the FPGA; the entire software application is operated and controlled at the ASIC, and the device-specific hardware is configured at the FPGA. Although FPGAs offer some flexibility, they are difficult to apply to IoT devices because they require physical access for reprogramming. Storing the synthesized hardware module in the device’s memory allows reprogramming without physical access, but it increases the memory size in a way that also increases the power consumption. Due to the limited memory size, it is not possible to prepare a large number of hardware modules, which makes it impossible to guarantee high flexibility.

Hardware-as-a-service (HaaS), which shares hardware in the cloud, has been studied to increase flexibility for hardware at the IoT device [16]. HaaS is a system that allows remote hardware devices distributed in various regions to be easily accessed through cloud middleware. Using HaaS, the edge device does not require additional hardware for accelerator, providing unlimited kinds of hardware services. However, to use remote hardware, data to be processed must be transmitted through the cloud middleware as in the operation of cloud computing. It makes the response speed of the system dependent on network latency. Also, because the hardware is attached to other devices, only general hardware, not edge-specific hardware, can be used.

**Figure 2:**
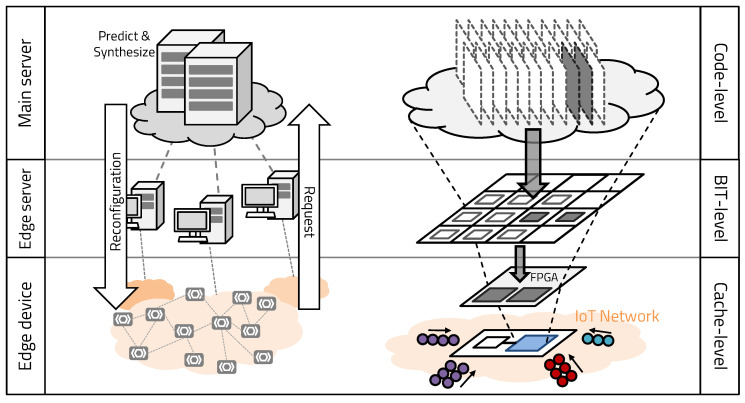
mIoT’s overall structure.

This paper extended from our previous work. Our first research represented our initial concept as a reconfigurable fault-safe processor platform [17]. Our second approach related to on-demand software replacement represents the possibility for real-time on-demand hardware execution [18]. In this paper, by integrating two approaches, we propose the metamorphic IoT (mIoT) platform, based on the flexible operation of cloud computing, with the powerful operation using hardware accelerators and real-time processing through the network independence of edge computing, as shown in Figure 2. Unlike previous research that sent data to be processed at the server, mIoT platform receives a hardware configuration to accelerate data processing from the server. By generating the hardware bitstream using the configuration parameter of the edge, the edge device can use edge-specific hardware. The method of transmitting the necessary hardware accelerators allows the edge device to enhance processing speed without network dependencies and gives the hardware the flexibility to cope with many situations. In addition, since the functions of various hardware can be executed in a small reconfigurable region, the overall chip size can be reduced, resulting in a significant reduction in power consumption. When reconfiguring the hardware, only a part of the FPGA is reconfigured, so the hardware reconfiguration and the program execution are processed simultaneously. The callability-based hardware prediction system minimizes time overhead by pre-reconfiguring the next required hardware, according to the program execution flow. Therefore, mIoT can rapidly process various data by reconstructing, in real time, diverse hardware functions, required in different environments on edge devices with limited hardware size.

## 2. Background

### 2.1. Dynamic Partial Reconfiguration

ASIC is a custom-designed integrated circuit chip, so its chip area is small, and the program execution speed is fast, resulting in low power consumption. However, once manufactured, ASIC cannot be changed, so it can only be used for single purpose. Recent IoT environments require a lot of functions. To accelerate all the functions with ASIC, the chip size and power consumption are increased, due to the hardware size of each function. We used a FPGA to provide various functions required by the IoT environment. The FPGA, which consists of reconfigurable gates, allows the user to reconstruct the circuit design by changing the connection of the gates using a hardware configuration tool.

The program consists of the functions that are constantly used and functions that are used at specific events. The edge must always have the hardware that accelerates the constantly used functions; other hardware is only needed during execution. Reconfiguring the entire FPGA consumes significant energy and time. To reduce unnecessary energy consumption and reconstruction time, we adopted partial reconfiguration technology that reconstructs only the part of the circuit at the FPGA. Dynamic partial reconfiguration (DPR) has also been developed, which can reconfigure in real time without the FPGA tool of the host computer [19,20].

**Figure 3:**
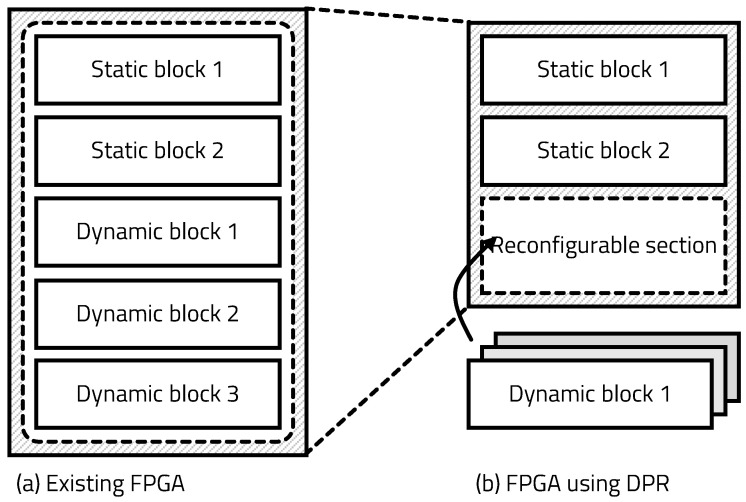
Blocks configured in existing FPGA and FPGA using dynamic partial reconfiguration (DPR).

Figure 3 shows the blocks configured in the existing FPGA and FPGA using DPR. The FPGA using DPR consists of a static region with fixed blocks and a dynamic region that can be reconfigured. To construct a conventional FPGA, a bitstream for the entire circuit is generated. However, using DPR, several bitstreams are created with a static module (SM), implemented at the static region, and a reconfigurable module (RM), implemented at the dynamic region. The bitstreams of each RM are implemented in the dynamic region according to the signal of the partial reconfiguration controller. Even if the RM is reconfigured, the SM in the static region is neither initialized nor stopped from executing. The DPR supports a variety of functions by reconfiguring the dynamic region as needed, but it does not use much area, so the required FPGA size is small. Due to the small size of the added FPGA, the area of the chip and the number of gates are reduced, thereby reducing power consumption. In addition, DPR provides flexibility in the selection of algorithms and protocols, because real-time circuit reconfiguration is enabled by configuring only a portion of the circuit.

### 2.2. RISC-V Processor Design Based on Chisel

**Figure 4:**
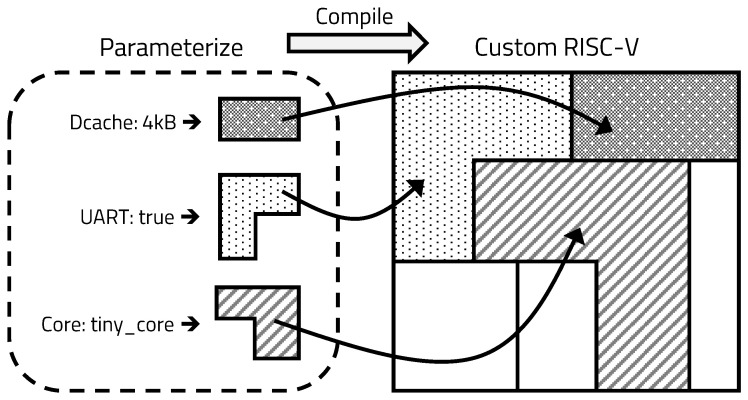
Processor design through hardware block parameterization using Chisel.

IoT devices with various constraints operate in a variety of application fields. To achieve optimal performance in each environment, the device must be designed for each field. Designing the hardware with a general hardware description language makes it harder to modify the design. As such, IoT devices with a universal design currently operate inefficiently in various environments. To solve this problem, we adopted building hardware in a constructing hardware in Scala-embedded language (Chisel) and RISC-V architecture in this paper [21]. Chisel is an open-source hardware construction language developed at UC Berkeley, which supports advanced hardware design, using highly parameterized generators and layered domain-specific hardware language. RISC-V is an open-source instruction set architecture (ISA) based on the reduced instruction set computing principles. Using Chisel to design hardware by parameterizing the constructs, we can efficiently configure the hardware to be optimized for a specific application, as shown in Figure 4.

### 2.3. Metamorphic IoT (mIoT) Platform

As IoT technology gradually develops, many functions are required at the edge and the functions of each given device must be managed continuously. At the general edge device, the processing unit is designed as an ASIC, and it performs simple operations and controls. When the edge demands to execute complex processing, the edge requests processing at the cloud computing server. As more and more devices are connected to the IoT system, requests to the server cause a bottleneck phenomenon in executing the functions, making it difficult to guarantee real-time performance. It seems like an attractive approach to adopt edge computing that affords the edge data processing capability to resolving this problem. However, due to the rapid development of IoT technology, the functions inside the edge also change quickly. Thus, the edge devices must insert only general-purpose hardware or replace the hardware at a short cycle to support the latest functions. Old devices that are not constantly maintained can become zombie devices, affecting the entire system. In this paper, we propose a mIoT platform to support and manage the amount of functions easily.

**Figure 5:**
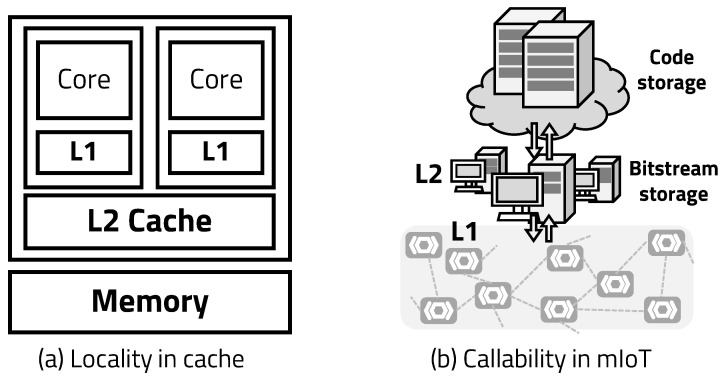
Similarity between callability and locality.

The “metamorphic” of mIoT refers to the fact that the internal structure and form change according to the external environments. The proposed mIoT is reconstructed with appropriate hardware, according to the external environments in which the device operates and the state of the embedded software. The mIoT consists of edge devices that execute applications and a server platform that manages and reconfigures the edge devices efficiently. The edge device relies on an ASIC-FPGA co-design architecture, which reconfigures hardware by receiving function blocks in real time from the server, according to the surrounding environment and the state of embedded software. The server uses a callability-based bitstream caching algorithm (BCA) to reduce the hardware reconfiguration overhead of the edge. We adopted the concept of callability from spatial locality, a characteristics of cache behavior in a typical processor, as shown in Figure 5. Spatial locality refers to the fact that if a particular memory space is referenced at a specific time, then the nearby memory space tends to be referenced in the near future. Dynamic region of mIoT is reconstructed with the RM determined by the operation of the processor, which is changed by the embedded application. As the application operates according to the control flow of the program, it is possible to predict that the function has the highest callability after a function is executed. Similarly, the RM can statistically predict the module to be called next according to the control flow. In this context, callability refers to the probability of which module may be called after the current operation. In this paper, we propose an ASIC-FPGA co-designed system that provides better flexibility to process data in various environments than a general processor and an accelerator system. It can reduce the program execution bottleneck on the server and the communication overhead in the IoT system.

## 3. Proposed Architecture

### 3.1. mIoT Edge Device

**Figure 6:**
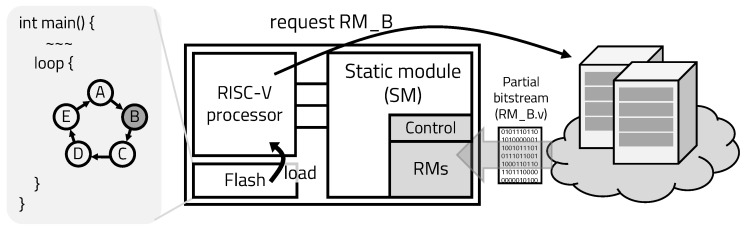
mIoT edge device structure.

Figure 6 shows the structure of the edge device in the proposed mIoT platform. The edge device is a co-design architecture that consists of an ASIC that acts as a processor, an FPGA that acts as an accelerator, and an external flash memory that stores embedded applications. The ASIC is a RISC-V architecture-based processor. The FPGA is divided into a dynamic region and a static region by applying the DPR, so it reconfigures the dynamic region by requesting in real time at the server, according to the operation of the application.

As mentioned above, ASIC is good, in terms of operating speed, power consumption and area, but it cannot be modified once it is manufactured. To accelerate the program while maintaining the advantages of the ASIC, it is important to separate the SM part from the RM part. In this case, study, the metamorphic fault monitor implemented on the FPGA observes the RISC-V processor implemented on the ASIC. To observe various points of the ASIC processor, the metamorphic fault monitor was reconfigured in real time. In this paper, we adopted the Freedom E300 platform, which is an open-source hardware based on RISC-V architecture and managed by SiFive, as an ASIC processor. The Freedom chip platform is designed using Chisel. Chisel parameterizes each hardware component and compiles each module into Verilog description language. The criteria for adopting the processor of the mIoT platform are as follows.

#### 3.1.1. Easy to Re-Design

As the IoT trend changes, hardware must be changed for devices optimized for various environments and operations. If the processor is designed only for a specific operation, power consumption increases, due to unnecessary hardware modules required when operating in different environments. Chisel makes it easy to modify the entire design to generate efficient hardware for various environments by objectifying the hardware with high-level descriptions and parameterizing the specifications of the hardware modules.

#### 3.1.2. Ownership of Design

Even if the processor is modified as required by the designer, it cannot claim to own the design. Such companies as SiFive (Freedom), Cadence (Tensilica), and Synopsys (ARC) have their architecture and they commercialize platforms that can create hardware designs based on their respective architecture. However, this method can only use the hardware provided by the company, but cannot be customized by the user. For copyright reasons, we used the RISC-V architecture of the open-source ISA.

#### 3.1.3. SW/HW Integrated Platform

To create a program that can be executed on a custom processor, we required processor-specific software build tools, such as a compiler, a linker, and a locater. The Freedom platform makes it easy to create hardware-optimized software, because it builds the processor-optimized build tools when building the processor.

### 3.2. mIoT Server

The mIoT platform has the advantage of being able to reconfigure the edge device in real time upon request. However, to guarantee real-time program execution at the edge, it is necessary to manage the time required for hardware reconfiguration. Therefore, the mIoT server must be managed to minimize the overhead of transmitting and reconfiguring hardware required for multiple edges.

**Figure 7:**
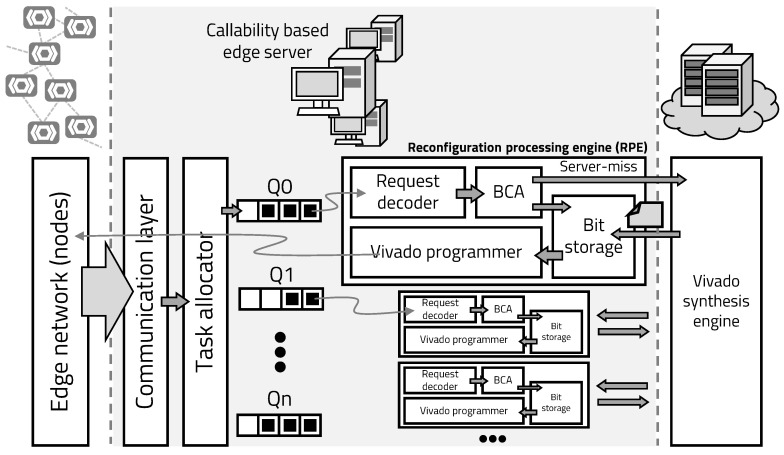
mIoT server structure.

Figure 7 shows the overall operation of mIoT platform. The mIoT server consists of edge servers that connect a group of IoT devices and the main server that connects edge servers. The task allocator receives the reconfiguration requests, sent by the IoT device, and assigns it to the queue of each reconfiguration processing engine (RPE). The RPE consists of a Vivado programming engine that can implement hardware into the edge’s FPGA, a BCA unit that manages the hardware to be reconstructed using callability-based prediction, and a decoder that interprets the request. While the embedded program is running, the edge’s hardware is pre-programmed based on callability. When the pre-programmed hardware is hit, that is expressed by edge-hit, and the MCU of the edge can use the accelerator directly, so the time overhead required for hardware is not required.

If the pre-programmed hardware is not hit, that is expressed by edge-miss, and the edge device asks the edge server to reconfigure the desired hardware. The transmitted reconfiguration request is allocated to the idle queue by the task allocator. The decoder fetches the queued request and decodes the necessary hardware information and the identification of the edge node. The BCA uses the decoded information to determine which hardware to reconstruct. If the bitstream of hardware being reconfigured is in the edge server’s bit storage, as denoted by server-hit, the IoT device is immediately reconfigured using the Vivado programming engine.

If the required bitstream is not in its storage, the edge server requests the required hardware from the main server, and the main server synthesizes the required hardware and transmits the bitstream. While the bitstream is implemented and executed at the edge device, the main server predicts the hardware to be used next, based on callability, and generates bitstreams and stores them in the edge server’s storage. In the mIoT platform, edge devices have very little space to store hardware bitstreams. The edge server has more storage than the edge device, and the main server has more storage than the edge server. To allow access to multiple bitstreams at the edge device with the least amount of time overhead, the mIoT platform uses a cache replacement algorithm, least recently used.

The Algorithm 1 represents the operation of the edge server according to the operation of the device. When the execution of $RM_{prog}$ is finished, the edge server programs $BIT_{next}$, which is a predicted bitstream, to RM. If the pre-programmed $RM_{prog}$ is not the desired hardware, the edge server requests the desired hardware with $E_{recon}$ signal. If the desired hardware, as denoted by $BIT_{req}$, is in the edge server’s storage, program the $BIT_{req}$ in the RM. In the absence of $BIT_{req}$, the edge server requests hardware synthesis from the main server and programs $BIT_{req}$ in RM. While the programmed $RM_{prog}$ is running at the edge, the edge server updates $BIT_{next}$ and $BIT_{stored}$.

Bitstream generation flow is shown in the Algorithm 2. On the main server, programs that are independent of the properties of the edge device are pre-synthesized. If the requested hardware is in the set of pre-synthesized code, the program is transmitted to the edge server and immediately programmed at the edge. To generate the bitstream optimized for the edge, the optimized Verilog code is synthesized and implemented. The generated code is transmitted to the edge server, programmed at the edge, and the main server predicts the next bitstream to be executed according to the caching algorithm, and synthesizes it to update the $BIT_{stored}$ of the edge server.
**Algorithm 1:** Edge bitstream caching algorithm.
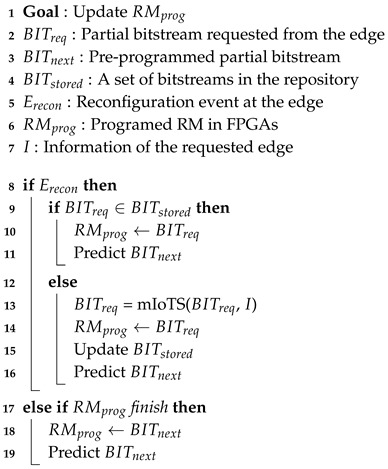


**Algorithm 2:** Generation flow of partial bitstream

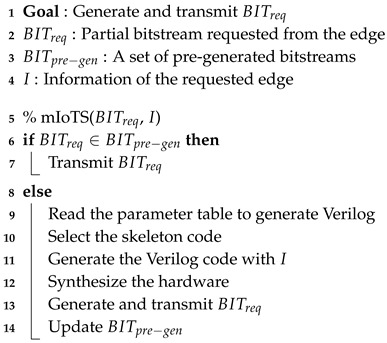



**Figure 8:**
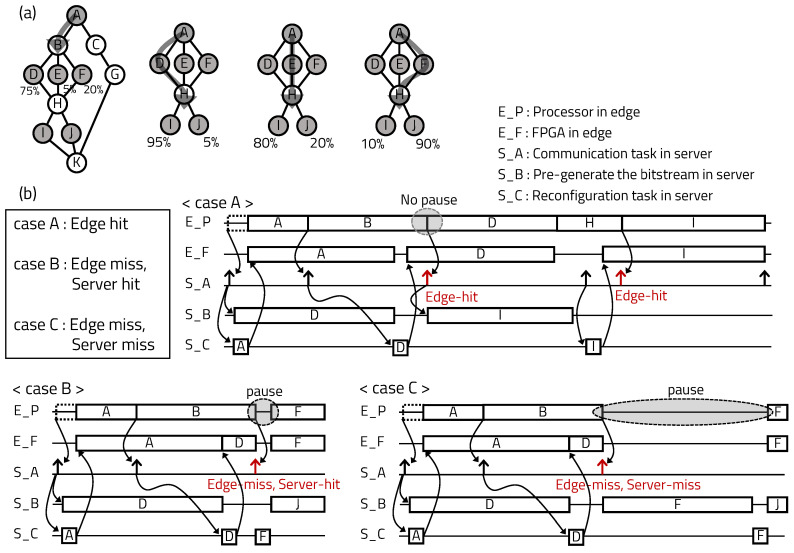
Case-by-case operating scenario of the proposed server system with a single RM. (**a**) Callability scenario from task perspective. (**b**) System operation flow by case.

Figure 8 shows the overall execution scenario of each case that can occur in the proposed mIoT platform. Figure 8a shows an example of callability from the perspective of tasks requiring hardware reconfiguration. When task B is executed, the callability of tasks D, E, and F being called are 75%, 5%, and 20%, respectively. After task D is called, the callability that task I and J will be selected next to task H are 95% and 5%, respectively. Each path has a different callability. Figure 8b shows the operation scenarios of the server, according to given example.

Case A shows the example of edge-hit with pre-programmed hardware based on callability. When the application starts to execute, the RM A stored on the edge server is reconfigured on the FPGA. The edge server requests and generates the RM D with the highest callability from the main server. When task A ends, the edge server pre-programs the prepared RM D into the FPGA. If the processor calls pre-programmed task D after task B, an edge-hit occurs and the edge device executes the application without time overhead due to reconfiguration.

In case B, edge-miss occurs because the pre-programmed RM D does not match, but server-hit occurs because the requested RM F exists in the storage of the edge server. If edge-miss occurs after task B is finished, the edge server searches the requested hardware at the storage and the FPGA is immediately reconfigured. In case of server-hit, the processor pauses due to the time overhead of partially reconfiguring the FPGA during application execution.

In case C, the bitstream that was previously generated by the edge server was also not hit, as expressed by server-miss. When server-miss occurs, the edge server requests hardware generation from the main server and receives it to reconfigure the FPGA. The main server generates bitstreams by synthesizing and implementing Verilog code. Then the processor takes a long overhead, because the requested hardware must be generated from Verilog code and wait to be reconfigured in the FPGA. After the FPGA is reconfigured, the edge server and main server update the bitstream set in the storage, according to the callability.

The mIoT server can perform synthesis and reconfiguration in parallel, in response to multiple requests. The bandwidth of tasks that can be executed in parallel depends on the server’s specification. If the number of requests exceeds the bandwidth, each request is managed by the task queue. Also, as shown in Equation (1), the server-miss reconfiguration overhead occurs for each device at the beginning of the operation, and consequently, the reconfiguration overhead converges to the edge-miss overhead. The edge-miss frequency can be reduced if the edge-hit frequency is increased which requires increasing the observation depth for determining callability. By combining the above operations, it is possible to ensure real-time performance by reducing the overhead required to reconfigure edge devices and effectively manage multiple IoT edge devices.

(1) $$T_{AVR}=\lim_{n\to \infty }\frac{T_{Server-MISS}+nT_{Edge-MISS}}{n}=T_{Edge-MISS}$$

where:

*n* = Number of executions

$T_{AVR}$ = Average overhead for reconfiguration

$T_{Server-MISS}$ = Reconfiguration overhead according to server-miss

$T_{Edge-MISS}$ = Reconfiguration overhead according to edge-miss

## 4. Case Study

### 4.1. Signal Processing (AI, DSP)

The first application that can apply mIoT is signal processing, such as artificial intelligence (AI) and digital signal processing (DSP). In signal processing, there are many operations to process the data that are received from the server, and to process such complex instructions as matrix multiplication and fast Fourier transform (FFT); the edge’s data are mainly transmitted to the cloud server for processing. However, the edge must be able to handle complex operations according to the demands of distributed processing such as edge computing. According to this trend, the edge addresses its lack of computational power by using a hardware accelerator, but it is not sufficient to perform all necessary operations at the edge. In addition, the accelerator of the edge has a fixed function and cannot be changed when it is designed as an ASIC; when it is designed as an FPGA, it is difficult to guarantee real-time performance, due to the overhead involved with reconfiguring the hardware. The mIoT predicts the next accelerator based on callability, and partially reconstructs the hardware in the FPGA. An edge device with mIoT can accelerate various functions in real time as if it has multiple accelerators and a small network dependency, compared to cloud computing.

### 4.2. Fault Monitoring

The second application that can apply mIoT is fault monitoring in IoT. IoT devices perform a variety of tasks in unsuitable environments and are exposed to many risks [22]. In addition, given the generalization of IoT, connected devices are rapidly increasing and connectivity is complicated, so the time in which a fault occurring in one node propagates to the entire system is accelerated. Therefore, it is important that each IoT device maintains stability [23,24]. To maintain the stability of the edge, a redundancy circuit is added at the critical part of the device; alternatively, the monitoring circuit watches periodically. The critical part is changed according to the surrounding environment and operation of the IoT device, so the monitoring object should be continuously changed to enhance stability. The existing fault tolerance circuit, made of ASIC, cannot change its hardware function, so it uses software to monitor flexibly. The monitoring software is executed by the processor, which affects the operation speed of the main application and cannot detect faults that occur below the clock level. Fault monitoring using mIoT increases the stability of the IoT system, because the monitoring object can be changed by reconfiguring a part of the hardware, according to the operation of the device.

## 5. Implementation

### Metamorphic Fault-Safe Processor (mFSP)

**Figure 9:**
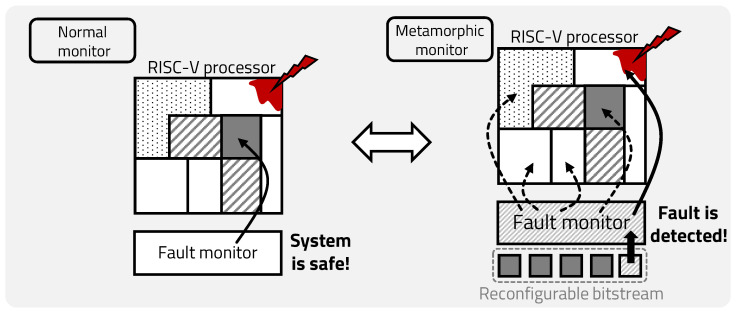
The need for metamorphic monitoring.

IoT devices that construct a large-scale IoT system have heterogeneous characteristics and are irregularly connected, which means that a malfunction in a single edge can affect the entire system. Moreover, IoT devices are exposed to various risks in a variety of environments, resulting in less stability. Technology research to maintain IoT has been conducted in many fields; for example, such research mainly uses techniques that compare processing results, using the duplicate and comparison technique for critical areas of hardware, and add redundancy circuits through voting techniques that maintain reliability [25,26,27]. In addition to the technology that uses hardware, the technology that uses software has developed as well. However, hardware technologies require an area cost, due to additional redundancy circuits, and software technologies cannot find clock-level faults. As shown in Figure 9, the normal fault monitor can only see pre-specified points, so it cannot find numerous faults of the edge occurring in various environments. To increase the stability of the device under these constraints, it is necessary to be able to flexibly detect various points, at a small additional area cost, using a metamorphic monitor.

**Figure 10:**
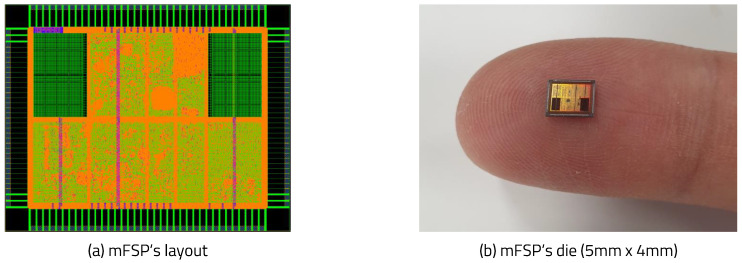
mFSP layout.

In this paper, we present the metamorphic fault-safe processor (mFSP) platform (Hynix/Magnachip, Icheon, South Korea) as a case study of the mIoT platform. We designed a chip optimized for each IoT edge with a Chisel-based RISC-V processor. The proposed FPGA prototype was implemented and verified on the ASIC chip. The ASIC was implemented with a Hynix/Magnachip 350 nm complementary metal-oxide semiconductor (CMOS) process. The chip layout is shown in Figure 10: the die size is 5 mm × 4 mm; the operating frequency is 25 MHz; and the number of logic gates excluding memory is about 110,000.

**Figure 11:**
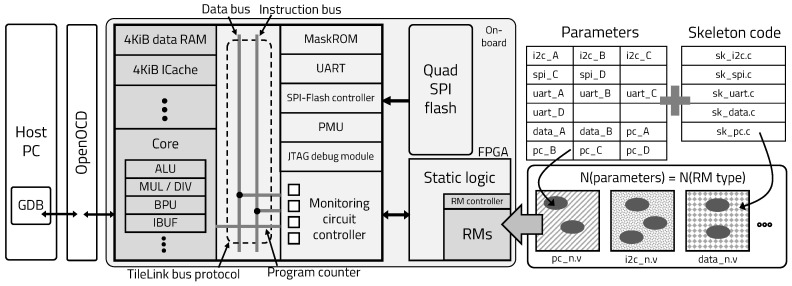
mFSP’s structure.

Figure 11 shows the structure of mFSP based on Freedom E300 platform, managed by SiFive with RV32IMAC ISA, 4KiB instruction cache, and 4KiB tightly integrated data memory. The mFSP structure includes UART hardware blocks for external communication and a controller for quad serial peripheral interface (SPI) flash, called code memory. It also consists of a mask ROM to execute the boot sequence, a power management unit (PMU), a joint test action group (JTAG) debug module to debug the operation and upload software, and a monitoring circuit controller to select signals from the processor and export the selected signals outside the processor. The user can debug the processor and download the software to the SPI-flash memory using GNU debugger (GDB) and openOCD.

**Figure 12:**
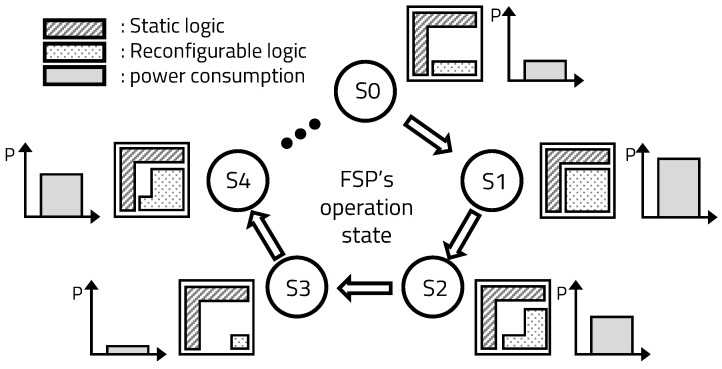
Power consumption optimization through real-time reconfiguration.

The FPGA consists of a static area and a reconfigurable area. The Verilog code, which is created by combining application-specific parameters and skeleton code on the server, is implemented in the reconfigurable area of the FPGA at the request of the processor. The mFSP reconfigures circuits that monitor different sections in real time to flexibly change critical sections, according to the operation of the processor, to maintain the stability of the device and the entire system. In addition, only parts necessary for program execution are used, because the hardware reconfiguration requires that the power consumption be reduced to execute the program, as shown in Figure 12.

## 6. Experiment

**Figure 13:**
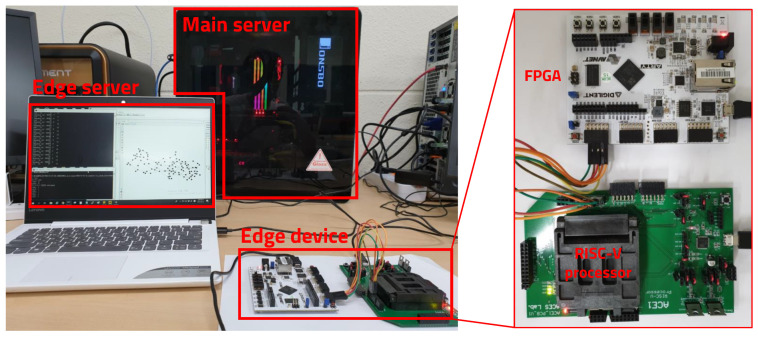
Experiment environment.

To verify the mIoT platform proposed in this paper, we combined the RISC-V processor implemented by a Hynix/Magnachip 350nm process and the Xilinx Arty-7 35T FPGA (AVNET, Tokyo, Japan), which can be partially reconfigured in real time to construct the mFSP edge device presented in the case study. As shown in Figure 13, The main server and the edge server for managing the reconfiguration of the edge device were constructed by the Windows Vivado environment. In the proposed platform architecture, the edge device and the server are connected wirelessly to manage the reconfiguration operation. Several studies have been published on wireless configuration of FPGA [28,29]; therefore, in the actual commercialization stage, we adopted the wireless configuration proposed in other studies and used JTAG configuration in this experiment.

**Figure 14:**
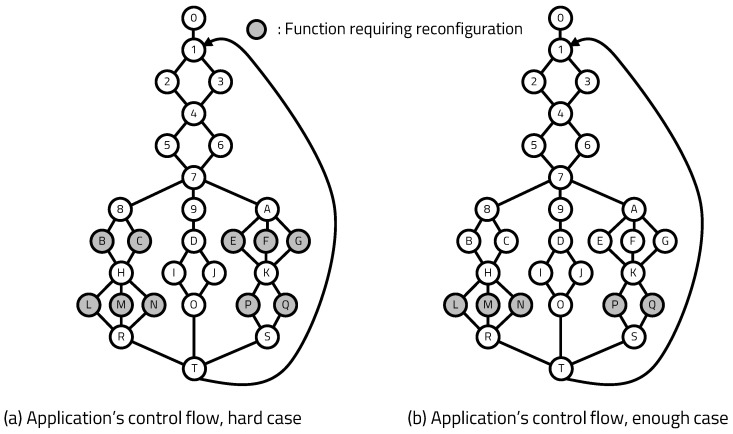
Application’s control flow.

The control flow of the software application to determine the operation of the processor is shown in Figure 14. The white circles in the figure are function blocks that operate software without the help of hardware, and the gray circles are function blocks that require additional hardware operation. When a gray block is executed, the processor requests the hardware to reconfigure the FPGA to the server, and the processor waits until the requested hardware is implemented. Each block that requires hardware has a certain level of callability, and caching operations for efficient hardware reconfiguration operate based on this callability. We designed the monitoring application to observe various parts of the FSP and presented difficult cases, in which hardware is called excessively, and enough cases that require one hardware call per execution.

**Figure 15:**
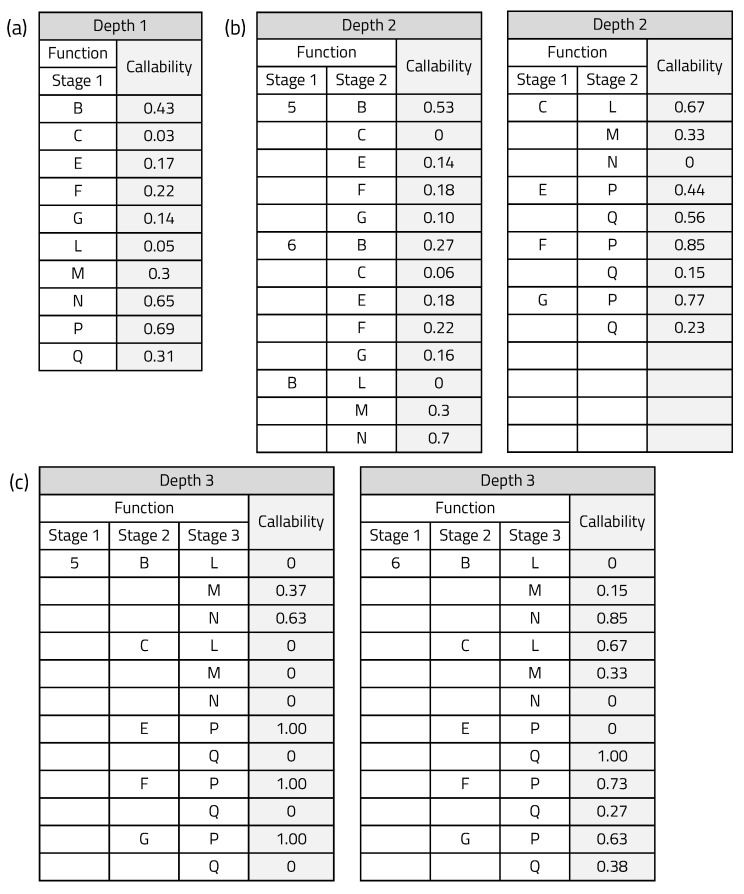
Callability extracted through iterative execution.

The callability of the application used in the experiment was determined by repeating an execution 1000 times, and the result is shown in Figure 15. The blocks that need hardware reconfiguration are B, C, E, F, G, L, M, N, P, and Q, and block 5 and 6 affect the callability of blocks that require hardware reconfiguration. Figure 15a shows the callability with an observation depth of 1, in which past execution does not affect current execution. Figure 15b shows the callability of a case with an observation depth of 2, in which only the immediately preceding execution affects the current execution. Finally, Figure 15c shows the callability with an observation depth of 3, observing up to two previous stages. Based on the callability shown in the above table, the BCA predicts the next hardware.

**Figure 16:**
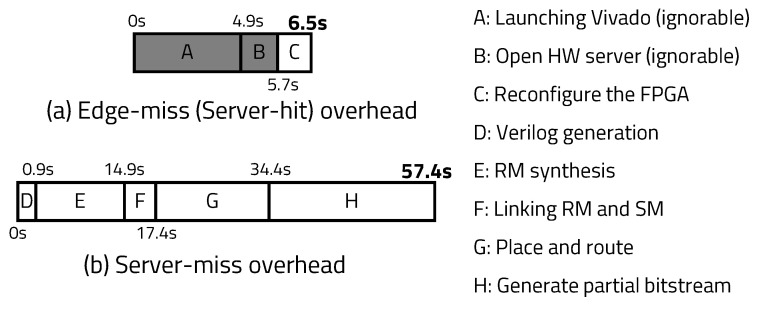
Overhead composition for each case.

As shown in Figure 16a, the average time required for hardware reconfiguration after edge-miss is 6.5 s. However, the actual time used to reconstruct the hardware is C, which is, on average, about 0.8 s. A and B represent the time required to start the platform, so these can be ignored in the context of long program execution. When server-miss occurs, as shown in Figure 16b, the server takes, on average, 57.4 s to generate, synthesize, place, and route (PNR) the Verilog code to generate bitstream. F is a step that consists of combining SM and RM, and if the module has previously been made, the D and E steps that create the module are not executed.

**Figure 17:**
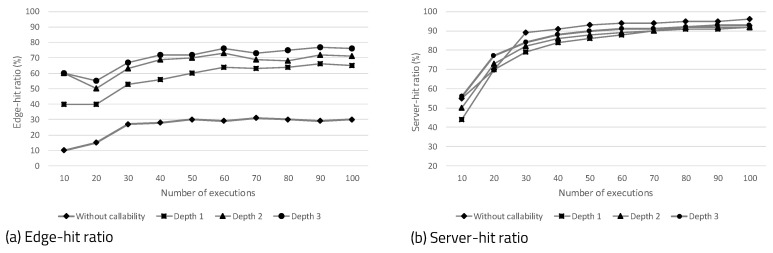
Hit ratio according to number of executions and observation depth.

Figure 17a shows the edge-hit ratio, according to the observation depth that determines the callability of the platform. The edge-hit is the most important factor, because it eliminates the time overhead involved in hardware configuration. We measured the edge-hit ratio at depths of 0, 1, 2, and 3, according to the number of executions of the program. The meaning of callability not being applied is that the caching algorithm arbitrarily determines the next hardware, considering only the operation sequence. For example, after B or C is executed, the callabilities of L, M, and N are 33%. Figure 17a indicates that the higher the observation depth, the higher the edge-hit ratio that can be obtained. Figure 17b shows the ratio of server-hit, according to the depth of observation. The server-hit occurs when the requested hardware is in the server’s bitstream storage. When the number of program executions is small, the server-hit ratio increases as the depth increases. However, if the program execution continues, the server-hit ratio becomes saturated, resulting in a similar value, regardless of depth.

**Figure 18:**
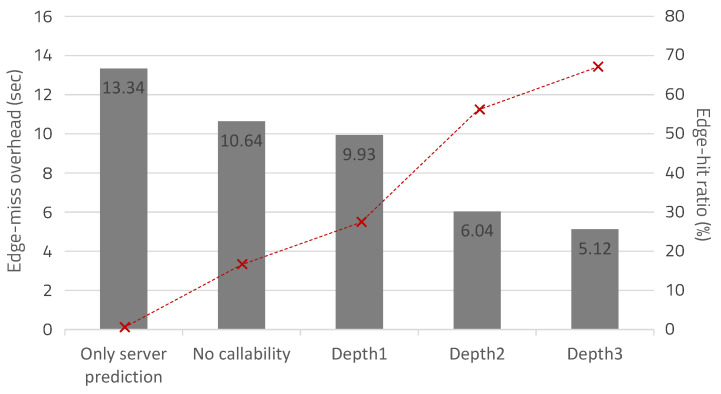
Overhead due to edge-miss according to the situation and edge-hit ratio.

Figure 18 shows the edge-hit ratio, according to the applied caching algorithm and the observation depth, as well as the time overhead when the hardware is reconstructed 18 times. This experiment was based on the assumption that the application has been sufficiently executed, so that server-miss does not occur. If edge caching was not applied, server-hit overhead was required for all reconfiguration operations, thereby requiring the largest reconfiguration overhead (13.34 s). When the caching algorithm was applied without callability, the edge-hit ratio was 16.67% and the reconfiguration overhead was 10.64 s. With an observation depth of 1, the edge-hit ratio was 27.78% and the overhead was 9.93 s. When the depth was 2 and 3, the edge-hit ratios were, respectively, 55.56% and 66.67%, and the overheads were, respectively, 6.04 s and 5.12 s. Therefore, the average overhead to reconfigure the hardware 18 times with an edge-hit ratio of 66.7% was 0.28 s.

**Figure 19:**
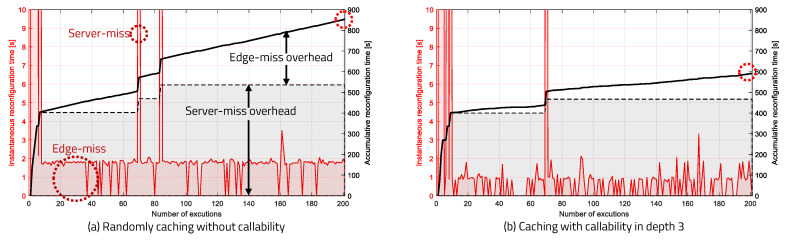
Instantaneous and accumulative reconfiguration overhead.

The frequency of occurrence of edge-miss and server-miss, according to the number of executions and the reconfiguration overhead, are shown in Figure 19: the red graph represents the instantaneous reconfiguration overhead; the black dotted line represents the accumulated server-miss overhead; and the solid black line represents the accumulated total overhead. The black dotted line shows that all instances of server-miss appear at the beginning of the run and no longer occur after the generation of all partial bitstreams. Therefore, we only must observe how frequently edge-miss occurs. We confirmed that more edge-miss overhead is required when edge caching and callability are not applied, as per Figure 19a, than when callability and edge caching are applied, as per Figure 19b. Also, when the observation depth is high, we confirmed a reduction in edge-miss overhead, according to Figure 19b.

**Figure 20:**
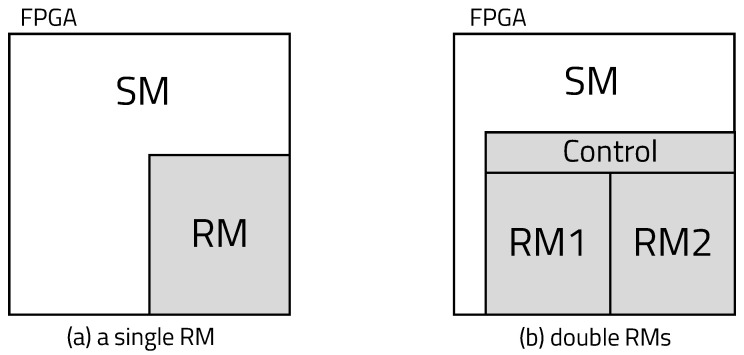
Experimental cases according to the number of RMs.

We conclude that a reconfiguration overhead of approximately 0.28 s per reconfiguration is required to achieve a 70% hit ratio, according to the results in Figure 18. However, this result can be seen as a reasonable reconfiguration overhead at a single-device level, but it is not reasonable for large-scale IoT systems. For example, edge-misses occur every cycle at 30% of the total nodes. For a system with 100 nodes, reconfiguration requests of 30 nodes are sent at one time to the edge server and the latency rapidly increases. Therefore, it is necessary to increase the hit ratio to apply the platform to large-scale IoT systems. Some ways to increase the hit ratio are as follows: observing more historical hardware call flows to accurately extract callability, and fetching multiple candidate RM modules to the edge device. In the previous experiment, we confirmed that the hit ratio is proportional to the depth of observation. In this experiment, we implemented two dynamic regions in the FPGA with a control block to increase the edge-hit ratio, as shown in Figure 20b. Both dynamic regions are reconstructed when edge-miss occurs. When one of the two reconstructed RMs is selected to start, the other region is reconstructed according to the new callability. In the structure with double RMs, we can apply Figure 14b, which is an enough-case application, in which reconfiguration occurs once per a single program execution cycle.

**Figure 21:**
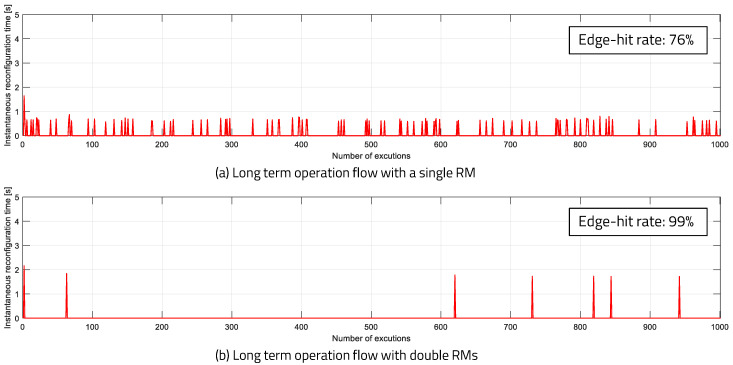
Long-term executions with Figure 14 flow.

**Figure 22:**
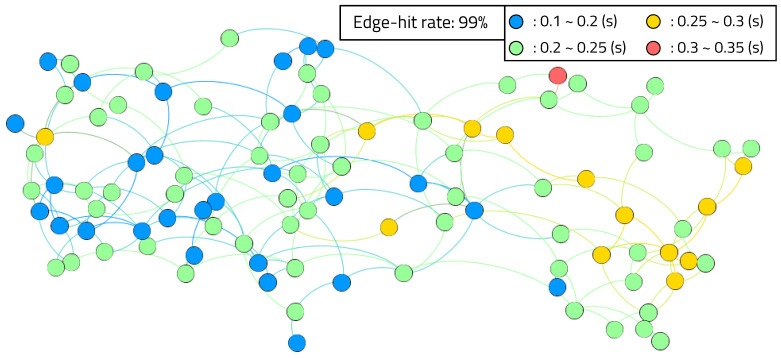
Large-scale simulation results with reconfiguration overheads.

**Figure 23:**
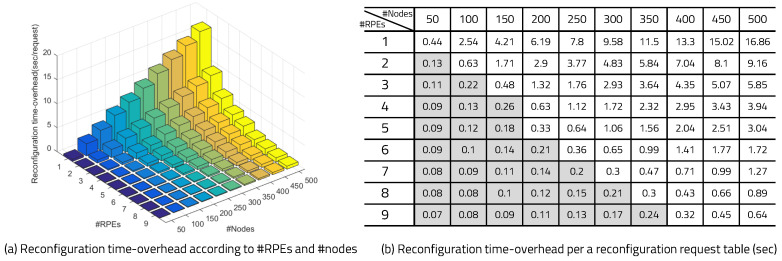
Reconfiguration overheads according to the number of reconfiguration engines and nodes.

Figure 21 shows the result of applying the double RM’s structure to a single device, as in previous experiments. In this experiment, we observed a long-term view, which consists of 1000 execution cycles after the program has been executed for a long time. As a result, the hit ratio with the single-RM case is 76%, and the hit ratio with the double-RM case is 99%. The results of a large-scale experiment, using 100 edge devices with 99% hit ratio and three RPEs, are shown in Figure 22. One device was implemented as an actual mIoT edge device, which consists of a RISC-V processor and FPGA, and the other devices were edge devices emulated by allocating applications in the edge server. Each device had zero to four reconfigurations during 200 executions, and the average reconfiguration overhead time was between 0.1 and 0.35 s. The reconfiguration overhead times for a given number of RPEs and a given number of edge devices are shown in Figure 23. The gray boxes indicate overhead results of less than 0.3 s. The number of RPEs and the number of edge devices connected to the edge network is determined differently, depending on the application.

Based on the above experimental results, it was possible to confirm that the proposed platform and the callability concept can manage the operation of each device with a reasonable reconfiguration overhead. Also, as the callability becomes more accurate, the correct RM candidate module is pre-programmed in the edge device, reducing the overall reconfiguration overheads of the IoT system.

## 7. Discussion

Real-time means that a program executing in a certain system guarantees a response within a specific time constraint. As a result of the experiment, the time required for hardware reconstruction using DPR is about 0.8 s, and the reconstruction overhead of mIoT using callability is 0.28 s and 0.2 s, respectively, when using single RM and using double RM. Because the proposed mIoT system requires some time for hardware reconfiguration, it is difficult to guarantee real-time performance in a delay-intolerant system. This paper reduces time overhead by increasing edge-hit by applying advanced callability algorithm and double-RM technology. This ensures real-time processing in areas such as intelligent home IoT, health care and wearable devices that are less sensitive to delays.

The mIoT in the intelligent home IoT field can speed up the processing of the edge IoT by learning the user’s life pattern with callability and pre-configuring the required hardware. Real-time in home IoT is based on human interaction, so the time overhead of 0.3 s is reasonable. In health care and wearable devices, data is pre-calculated at the edge and only necessary information is transmitted to the server, because personal data security is important. The mIoT edge improves computation speed by transmitting accelerators required for data computation. In this case, overall execution time is reduced even with the time overhead due to hardware reconfiguration.

## 8. Conclusions

In this paper, we proposed a metamorphic IoT platform that combines the following components and concepts to enhance stability in the diverse IoT operation environments and the processing capability at the edge: an ASIC-FPGA co-design-based edge device, able to provide various hardware with limited resources, a server system to manage a large number of edge devices connected to a network, and a callability to reconfigure edge devices with minimal time overhead. The IoT edge device is optimized for the operating environment, based on the Chisel language and RISC-V architecture, and the FPGA is partially reconstructed in real time, according to the operation of the processor. The server predicts the next hardware to be used in the edge, based on the callability, greatly reducing the time required to reconfigure the edge device. The proposed platform broadens the range of hardware that one edge device can use, and facilitates the management and update of reconfigurable hardware, thereby extending the operating life of a given edge device. In addition, it processes data using hardware accelerators at the edge, so the data transfer time is less than that of the existing platform, which makes the operation speed fast and allows for the resolution of the bottleneck of the server. With the experiment, which used actual devices and an interworking server, we confirmed that the overhead required for reconfiguration is reasonable and that the proposed callability-based reconfiguration system is efficient. Using the proposed platform, we can construct an IoT system that can guarantee flexible operation in real time in a complicated IoT environment.

This paper achieved real-time reconstruction of various accelerator in limited hardware size to increase the processing speed and reduce power consumption of the edge device. The mIoT platform has several security issues, such as man-in-the-middle attack and hardware Trojan, while transmitting hardware information for reconfiguration over the network. In the future, this study will be expanded to apply encryption for hiding bitstream from attackers.

## Abbreviations

The following abbreviations are used in this manuscript:

MCU	Micro Controller Unit
FPGA	Field Programmable Gate Arrays
ASIC	Application-Specific Integrated Circuit
BCA	Bitstream Caching Algorithm
RPE	Reconfiguration Processing Engine
UART	Universal Asynchronous Receiver Transmitter
